# Ossifying Parotid Carcinoma ex Pleomorphic Adenoma

**DOI:** 10.1155/2015/395358

**Published:** 2015-05-17

**Authors:** Suresh Mohan, Sidharth V. Puram, Bharat Yarlagadda, Vania Nosé, Daniel G. Deschler

**Affiliations:** ^1^The Warren Alpert Medical School of Brown University, Providence, RI 02912, USA; ^2^Department of Otology and Laryngology, Harvard Medical School, Boston, MA 02115, USA; ^3^Department of Otolaryngology, Massachusetts Eye and Ear Infirmary, Boston, MA 02114, USA; ^4^Department of Pathology, Massachusetts General Hospital, Boston, MA 02114, USA

## Abstract

We present a unique case of an extensively ossified carcinoma ex pleomorphic adenoma (CXPA) in a 76-year-old man with a five-year history of a slowly growing parotid mass. Fine-needle aspiration of the mass was nondiagnostic. A computed tomography (CT) scan of the lesion revealed a well-circumscribed mass with peripheral calcification. Initial pathological analysis suggested a benign parotid mass, but rigorous decalcification revealed noninvasive CXPA. The patient underwent complete resection of the mass and remained disease-free nine months later. Extensive ossification of a seemingly benign parotid mass may mask areas of carcinoma that may progress if left untreated.

## 1. Introduction 

Carcinoma ex pleomorphic adenoma (CXPA) is an infrequent but aggressive salivary gland malignancy that develops in association with a primary or recurrent benign pleomorphic adenoma (PA). Though its pathogenesis remains unclear, it is hypothesized to be the result of malignant transformation within a PA [1, 2]. Accordingly, CXPA can range from a pocket of malignant cells* in situ* to high-grade, frankly invasive disease with regional and distant metastases [2]. Diagnosis is difficult as the residual PA component is often small and overlooked, and the carcinomatous portion may be heterogeneous [2].

## 2. Case

A 76-year-old man presented with a five-year history of a right parotid mass that had slowly grown since it was first noticed. The patient denied any symptoms, including dysphagia, facial weakness, or otalgia. A prior fine-needle aspiration of the mass was nondiagnostic. Past medical history was notable for hypertension and prostatic carcinoma treated with brachytherapy. He used alcohol daily and was a former smoker. On examination, a 3.5 cm mobile mass in the tail of the parotid was identified. Facial nerve function was intact and symmetric.

**Figure 1:**
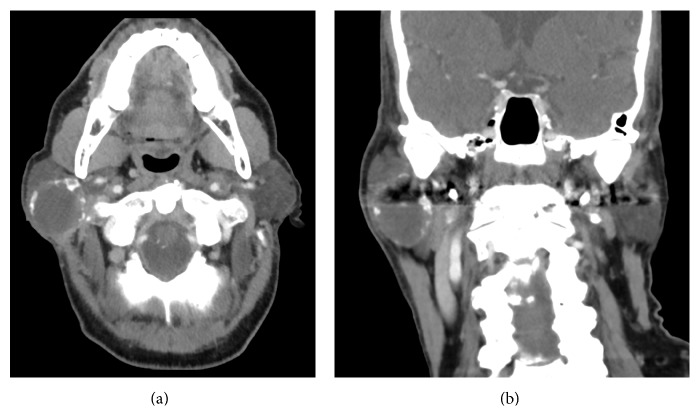
Computed tomography scan. (a) Axial and (b) coronal sections demonstrating a well-circumscribed, solid, isodense 4 × 3.5 cm mass with rim of peripheral calcification.

Computed tomography (CT) of the neck revealed a well-circumscribed, solid, isodense 4 × 3.5 cm mass, with no invasion into adjacent structures (Figure 1). The mass contained a rim of peripheral calcification with no internal calcifications. No lymphadenopathy was observed in the remaining neck.

**Figure 2:**
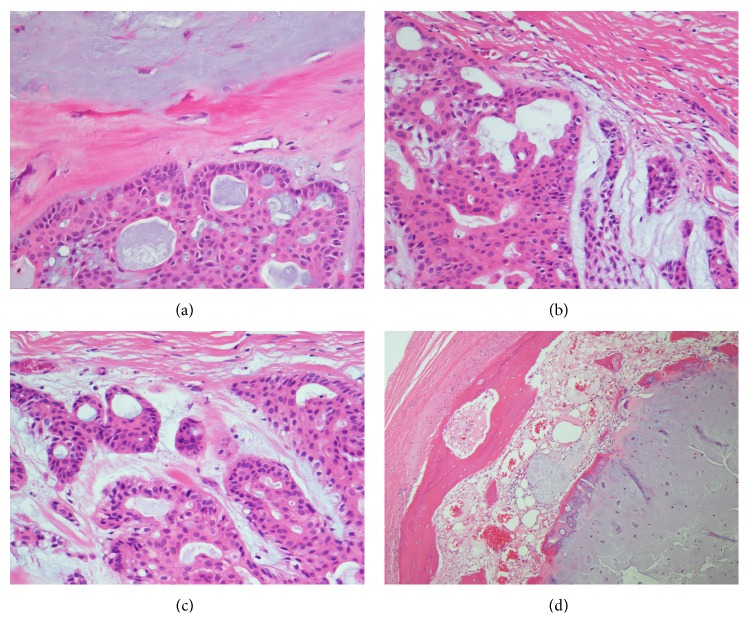
CXPA microscopic examination. (a) The benign myxoid component (top) is separated by a thick fibrous band from the malignant component (bottom). The neoplasm is composed of large cells with eosinophilic cytoplasm in a glandular pattern, H&E, 400x. (b) The well-circumscribed and encapsulated tumor shows areas of residual pleomorphic adenoma (lower right). A more cellular area (left) with irregular glandular architecture and composed of large cells with eosinophilic cytoplasm and pleomorphic nuclei is confined within the capsule, H&E, 400x. (c) The neoplasm is surrounded by a thin capsule (top) and cells show remarkable degree of cytological pleomorphism with hyperchromatic nuclei and prominent nucleoli, H&E, 400x. (d) The ossification comes from the periphery of the tumor within the capsule (left) and is present at the periphery of the benign components of the tumor (right), H&E, 100x.

The patient underwent right parotidectomy with facial nerve preservation. Grossly, the specimen contained a 4 × 3.5 cm well-circumscribed, tan-white, firm, and focally cystic mass containing yellow-green gelatinous material, a thick fibrous capsule, and significant calcifications. After decalcification, histopathology revealed elements of salivary acini, a stroma of variable chondroid and myxoid composition, and ossification of the periphery (Figure 2). Final pathological diagnosis was carcinoma ex pleomorphic adenoma (CXPA), noninvasive with extensive ossification. The patient did well postoperatively with no new complaints and remained symptom-free nine months later.

## 3. Discussion

CXPA is categorized as a mixed malignant tumor, but it is exceedingly more common than the other subtypes, carcinosarcoma and mixed metastasizing PA [2]. It is most commonly observed in patients in the sixth and seventh decades of life, and the male to female ratio has been reported as 1.18–2 : 1 [1, 2]. Image guided fine-needle aspiration cytology (FNAC) is commonly used in preoperative diagnosis but may be of decreased yield in such heterogeneous lesions due to sampling error [1, 3]. CT or magnetic resonance imaging (MRI) can be valuable in assessing the extent of local invasion, perineural spread, lymph node metastases, and parapharyngeal space involvement [3]. Unfortunately, due to the coexistence of multiple tissue types, CXPA imaging findings can be nonspecific and poorly distinguished from other benign or malignant salivary gland tumors. For example, PA and CXPA may appear similarly on CT, with calcified regions of cartilaginous elements seen frequently in both [4]. Kato and colleagues recently have suggested using diffusion-weighted MRI to distinguish benign and malignant tissue in CXPA [5]. However, regardless of the radiologic modality, as in the case of our patient, extensive ossification can potentially obscure the presence of a malignancy and must therefore be assessed carefully. Here, we provide a rationale for increased scrutiny of parotid tumors containing extensive ossification.

Ossification is a complex, incompletely understood process that depends on the local concentrations of calcium and phosphate, pH, calcium-binding protein, alkaline phosphatase, and proteoglycans [6]. Heterotopic ossification in tumor stroma is a well-known phenomenon in epithelial tumors of the breast, kidney, ovary, and digestive tract. In the head and neck, the tongue is the most common site, while, in contrast, parotid tumor calcification is rare. When present, however, ossification in parotid masses is highly suggestive of pleomorphic adenoma [4, 6]. PAs commonly contain mucoid, chondromyxoid, or hyalinized mesenchymal-like elements, but reports of extensive stromal ossification are limited [6–8]. Furthermore, whether this ossification is endochondral, originating from a cartilaginous precursor, or due to direct deposition by metaplastic myoepithelial cells within the tumor remains unclear. Multiple reports support both theories [7–9].

Reports of ossification of CXPA are extremely rare. Spencer et al. reported the first ossifying CXPA identified on CT, noting that punctate calcifications in both benign and malignant PAs had been observed previously, but without bone formation [4]. They describe a large parotid carcinoma with a core of well-formed, trabecular bone on CT, revealing its origin as a PA, but without any concealment of the carcinomatous portion of the mass. They also recommend CT over MRI as a modality for imaging PA, noting that MR is less reliable in assessing necrotic tumor foci, a potential marker of malignant change, and in identifying calcifications or tumoral bone [4]. In contrast, our case features a benign appearance on CT. The mass appeared congruent with surrounding parotid parenchyma with no invasion, necrosis, or lymph node involvement. Besides extensive peripheral calcification, there were no particularly remarkable features. The carcinoma was only discovered after careful pathologic analysis involving rigorous decalcification. This suggests that extensive calcification can mask pockets of carcinoma that may progress if left untreated. Since imaging is inherently limited in this context, a high degree of suspicion by the otolaryngologist, radiologist, and pathologist is important in diagnosing such occult carcinomas in the presence of extensive ossification.

The differential diagnosis of calcified parotid or parapharyngeal tumors is broad and includes pleomorphic adenoma, soft tissue chondroma or osteoma, extracranial meningioma, osteocartilaginous tumors arising from bone exostoses [7], vascular ossification of atherosclerotic lesions, or hereditary conditions such as Albright's syndrome and fibrodysplasia ossificans progressiva [6]. This case emphasizes the importance of clinical suspicion for a malignancy in the presence of an ossified mass. The extensive ossification yielded a benign radiographic appearance with no extra-salivary invasion, lymph node involvement, or necrosis, despite the underlying carcinoma. While the presence of ossified components in a parotid tumor is virtually pathognomonic of PA, it is important to remain vigilant for an occult carcinoma and perform thorough pathologic analyses.
